# Patient-Specific or Conventional Instrumentations: A Meta-analysis of Randomized Controlled Trials

**DOI:** 10.1155/2020/2164371

**Published:** 2020-03-10

**Authors:** Yipeng Lin, Wufeng Cai, Baoyun Xu, Jian Li, Yuan Yang, Xuelin Pan, Weili Fu

**Affiliations:** ^1^Department of Orthopedic Surgery, West China Hospital, Sichuan University, Chengdu 610041, China; ^2^Department of Radiology, West China Hospital, Sichuan University, Chengdu 610041, China

## Abstract

**Objective:**

To conduct a meta-analysis of randomized controlled trials (RCTs) to compare knee arthroplasty with patient-specific instrumentation (PSI) with the conventional instrumentation (CI).

**Methods:**

RCTs were selected in PubMed and Embase from 2012 to 2018. Key data extracted included malalignment of mechanical axis, blood loss, surgical time, Oxford Knee Score (OKS), Knee Society Score (KSS), length of stay, and complications. Subgroup analysis was also performed regarding different PSI systems and different image processing methods.

**Results:**

29 RCTs with 2487 knees were eligible for the meta-analysis. Results showed that PSI did not improve the alignment of the mechanical axis compared with CI, but MRI-based PSI and Visionaire-specific PSI decrease the risk of malalignment significantly (*P* = 0.04 and *P* = 0.04 and *P* = 0.04 and *P* = 0.04 and *P* = 0.04 and

**Conclusion:**

PSI reduced the blood loss and improved KSS. MRI-based PSI reduced operative time and risk of malalignment of mechanical axis compared with CT-based PSI. Moreover, Visionaire-specific PSI achieves better alignment result of the mechanical axis than other systems.

## 1. Introduction

Total knee arthroplasty (TKA) is the most important treatment for end-stage osteoarthritis of the knee and has been performed increasingly common in orthopedics surgery. Goals of this procedure include pain relief and alignment correction [1]. Promising outcomes has been reported by this procedure. Nevertheless, mechanical axis malalignment remained a problem, which may result in aseptic loosing, instability, and unexplained pain postoperatively [2, 3]. To achieve better anatomical and functional outcomes, patient-specific instrumentation (PSI) has been introduced.

Compared with conventional instrumentation (CI), PSI uses customized cutting blocks instead of standard blocks. The customized blocks were generated from a preoperative three-dimensional model, which was reconstructed from computed tomography (CT) or magnetic resonance imaging (MRI) [4]. Reported advantages of PSI included improved alignment, higher surgical efficacy, and reduced complication risk [5]. However, the superiority of PSI over CI remained inconclusive.

Previous meta-analyses demonstrated no significant difference between PSI and CI in short-term follow-up results, regarding postoperative functional outcomes [6, 7] and radiographic alignment [8, 9]. Mannan et al. [6] conducted a meta-analysis including five RCTs and three prospective-comparative studies that revealed no significant difference between PSI and conventional instrumentation for postoperative Knee Society Score (KSS), ROM, or Oxford Knee Score (OKS). Thienpont et al. [8] compared the mechanical alignment between PSI and CI, concluding that PSI might improve the accuracy of femoral component alignment and global mechanical alignment, but increased the risk of outliers for the tibial component alignment. However, most meta-analyses included nonrandomized studies and with small size of patients. Besides, they analyzed only a few variables (outliers from radiology only or functional outcomes only, etc.), which is not robust enough to determine the superiority of one over another. Furthermore, more randomized controlled trials (RCTs) have been published [10–15] recently and need to be integrated to update our knowledge (comprehensive comparison between our work and previous reviews can be found [Supplementary-material supplementary-material-1] Supplemental file).

We therefore conducted a meta-analysis, including all level one studies that are comparing the PSI and CI methods to treat osteoarthritis, to answer three questions: (1) Intraoperatively, do patient-specific instrumentations reduce the risk of blood loss and shorten the surgical time compared with conventional instrumentation? (2) Postoperatively, does the use of PSI yield improved radiological and functional results while reducing the risk of complication? (3) Integrating the above data with patients' length of stay in the hospital, does PSI provide financial benefit and achieve better cost-effectiveness results? Finally, we hypothesized that PSI did not improve the alignment of the mechanical axis or functional outcomes compared with CI.

## 2. Materials and Methods

### 2.1. Search Strategy

We employed a validated search strategy by using the Preferred Reporting Items for Systematic Reviews and Meta-Analyses (PRISMA) checklist. Two databases, PubMed and Embase, from 2012 to 2018 were searched.

The following terms including mesh terms and free terms for PubMed were used in the search strategy: total knee arthroplasty, patient-matched, custom-made guides, patient-specific, and custom-fit. Reference lists from relevant articles were retrieved to identify additional studies. We also complemented our reference by searching gray literature and unpublished literature database.

### 2.2. Study Selection and Eligible Criterion

2 reviewers independently screened all search terms, abstract, and full text of studies potentially eligible for including. A third senior reviewer was consulted when disagreements occurred.

For the types of participants, studies involving males or females of all ages were included.

The types of interventions and controlled groups were as follows: (1) the comparison between patient-specific instrumentation (PSI) and conventional instrumentation (CI) in the aspect of clinical, functional, or radiographic outcomes; (2) patients who underwent primary total knee replacement; and (3) studies that were published in English. Studies were excluded if they reported the patients with fracture, deformity, or tumor, and if they were animal or cadaveric studies.

For the types of studies, 29 studies included were all randomized clinical trials. No language, publication date, or publication status restrictions were imposed.

The types of outcome measures were operative time, blood loss, malalignment of mechanical axis, and Knee Society Score (KSS).

### 2.3. Data Extraction

We used a standard form to extract data, and after extracted by one investigator, the data would be verified by the other investigator. The data extracted was strictly confined in the same follow-up time. Data extraction included outliers of the mechanical axis, which was defined as a measurement of >3° on standing full-extremity radiograph in full extension [16], total operative time, blood loss (measured by volume loss or reduction of hemoglobin), and postoperative functional score, including Knee Society Score (KSS) and Oxford Knee Score (OKS). Subgroup analysis was conducted in the KSS-knee and KSS-function, the MRI-based and CT-based operative time and malalignment, and specific system of PSI. Besides, length of stay in hospital and complication rate were also extracted.

### 2.4. Quality Assessment

The methodological quality of included studies was assessed by 2 independent investigators. The *Cochrane Handbook for Systematic Reviews of Interventions* was used for assessing all randomized studies. Six items were examined to value the risk of bias: (1) random sequence generation, (2) allocation concealment, (3) blinding of participant and personal, (4) blinding of outcome assessment, (5) incomplete outcome data, and (6) selective reporting. Each item is classified as low risk of bias, high risk of bias, or unclear risk of bias.

### 2.5. Data Analysis

We used *P* value and *I*^2^ to evaluate the statistic heterogeneity, *P* < 0.1 and *I*^2^ > 50%, indicating high heterogeneity. The fixed effects model was conducted to the homogeneous data when *I*^2^ < 50%, while the random effects model was performed to the data with high heterogeneity. Revman 5.3 (Version 5.3. Copenhagen: The Nordic Cochrane Centre, The Cochrane Collaboration) was used for the data analysis. For dichotomous variables, risk ratio and 95% confidence intervals were worked by the Mantel-Haenszel method. Forest plot was used to present our statistic from PSI and CI groups. The Funnel plot was conducted to assess publication bias when variables were extracted from more than 10 studies. For studies that did not report the standard deviation (SD), we calculated it from *P* value, 95% confidence intervals, interquartile range, or standard errors. If this information was not mentioned, we will contact the corresponding author for the missing part.

## 3. Results

### 3.1. Search Findings

**Figure 1:**
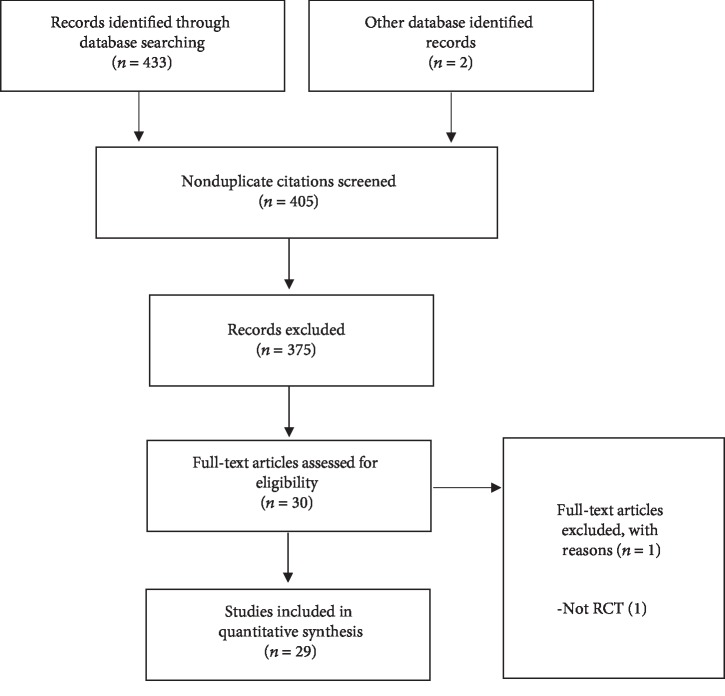
PRISMA flow diagram for selection of included RCTs.

The results of the search strategy are presented in PRISMA flowchart (Figure 1). A total of 433 studies were identified from the initial search. 29 studies, which are all randomized controlled trials (RCTs), met the final inclusion criteria for the meta-analysis. Study baseline characteristics are summarized in Table 1. Twenty-nine randomized studies representing 2487 TKAs (PSI: 1243, CI: 1244) were included.

### 3.2. Study Characteristics

All these included studies had level I evidence and were published between 2012 and 2018 (Table 1). The average follow-up time is 10.9 months. The average age of included patients is 68.3 years. For the PSI group, 21 studies acquired 3D models of the patients' anatomy from magnetic resonance imaging (MRI) while 8 studies chose computed tomography (CT) to acquire images. Signature is the most commonly used PSI system (8/29), followed by Visionaire, Zimmer PSI, and TruMatch (7/29, 5/29, 4/29, respectively). Myknee, Miscellaneous, Materialise NV, and Imprint were also used in the included studies. Most studies are based in Europe (20/29), followed by the United States (4/29), Asia (4/29), and Australia (1/29).

### 3.3. Quality Assessment and Risk of Bias

**Figure 2:**
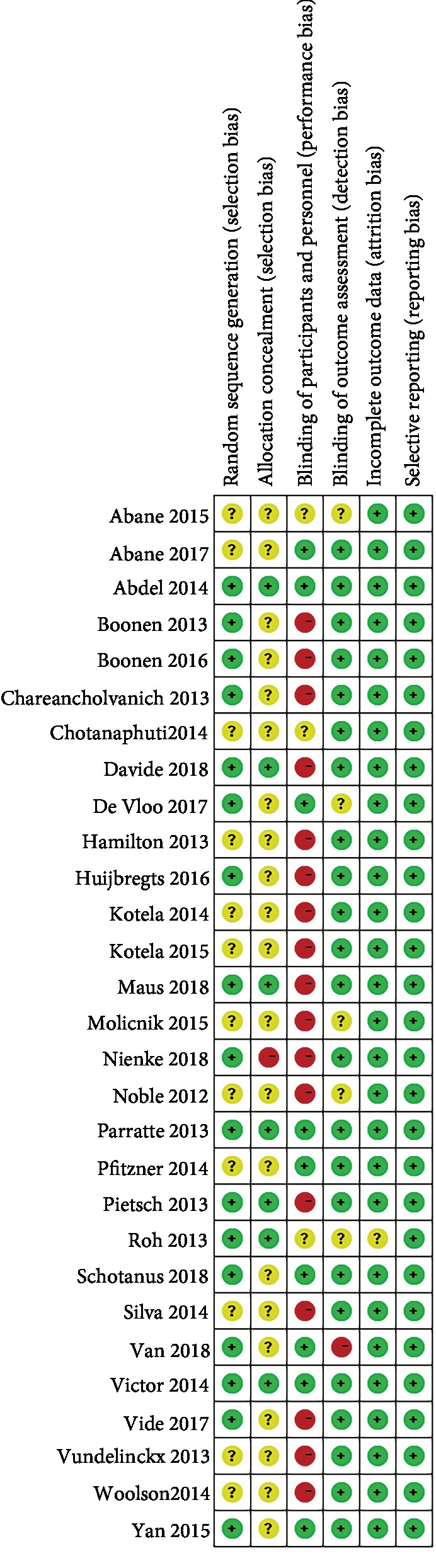
Risk of bias of included studies. + = low risk; – = high risk; ? = unknown risk.

All included RCTs were evaluated by the *Cochrane Handbook for Systematic Reviews of Interventions*. More than 50% of the included studies have a risk of performance bias. Nearly 75% included studies were not mentioned the allocation concealment, thus resulting in a selection bias. All studies were free of incomplete outcome data and selective reporting (Figure 2).

### 3.4. Operative Time

**Figure 3:**
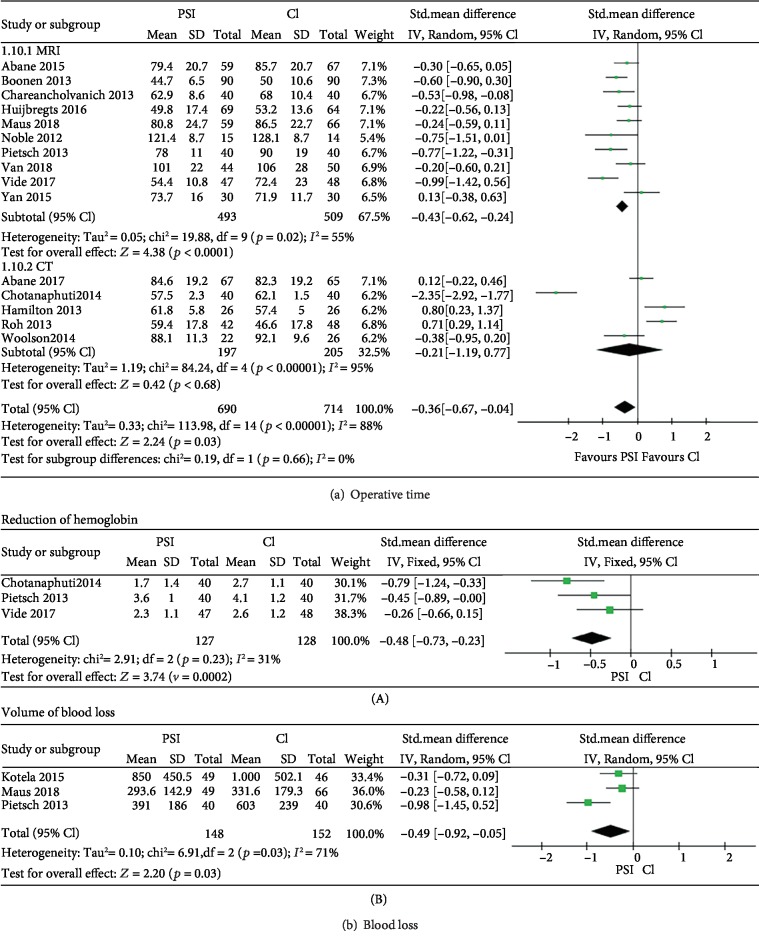
Forest plot operative time and blood loss. (a) Forest plot operative time, subgroup analysis by MRI-based and CT-based PSI. (b) Forest plot of blood loss evaluated by reduction of (A) hemoglobin and (B) volume of blood. PSI: patient-specific instrumentation; CI: conventional instrumentation; SD: standard difference.

Fifteen studies [10, 12, 15, 17–28] with 1,404 knees were included for the analysis of operative time. The total standardized mean difference (SMD) was -0.36 (95% CI, -0.67 to -0.04; *P* = 0.03) (Figure 3(a)). Substantial heterogeneity was found in the statistical analysis (*I*^2^ = 88%, *P* < 0.00001). Subgroup analysis of operative time between MRI-based or CT-based image processing favors the MRI group. Unlike the result from overall effect or from the isolated MRI-based group, the CT-based group showed insignificant difference regarding operative time between PSI and CI.

### 3.5. Blood Loss

Data of blood loss, from 5 studies, were pooled; 2 of the 5 measured the reduction of hemoglobin (g/dL) [17, 22], 2 studies measured the loss volume of 11 blood [12, 29], and one study [24] measured both. In both methods, we found a significant reduction of blood loss. The Std. Mean Difference was -0.48 (95% CI, -0.73 to -0.23; *P* = 0.002) and -0.49 (95% CI, -0.92 to -0.05; *P* = 0.03) for the hemoglobin loss and blood volume, respectively (Figure 3(b)).

### 3.6. Malalignment of Mechanical Axis

**Figure 4:**
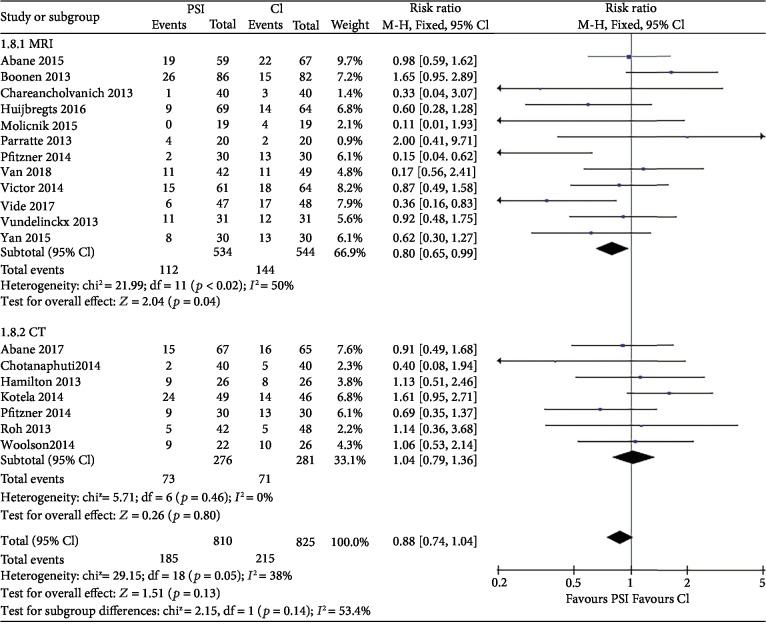
Forest plot of malalignment of mechanical axis. Subgroup analysis by MRI-based and CT-based PSI. Abbreviations: PSI: patient-specific instrumentation; CI: conventional instrumentation; SD: standard difference.

**Figure 5:**
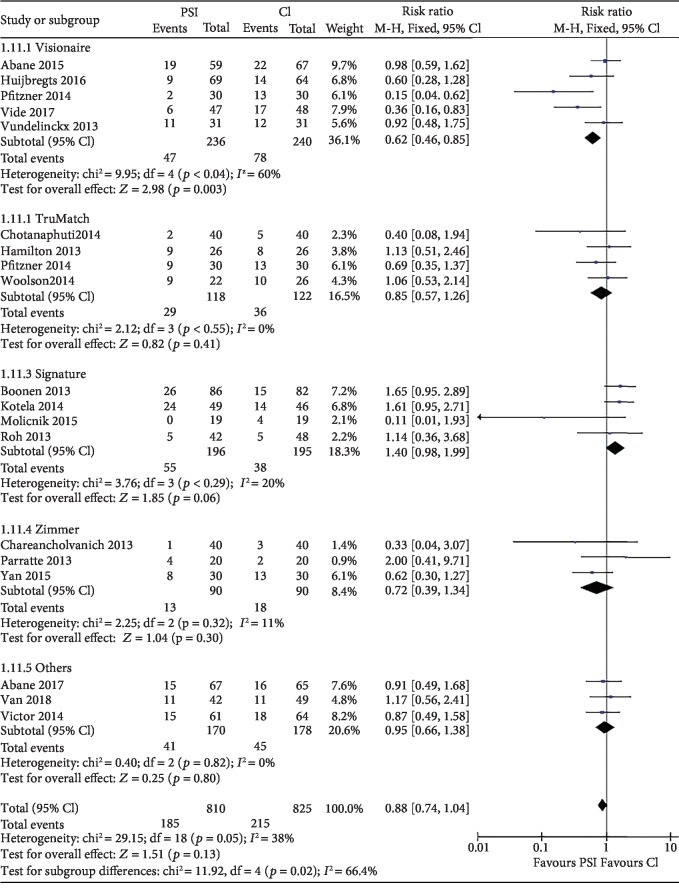
Forest plot of malalignment of mechanical axis. Subgroup analysis by system-specific PSI. Abbreviations: PSI: patient-specific instrumentation; CI: conventional instrumentation; SD: standard difference. Other PSI systems: a: MyKnee; b: Materialise NV; c: Miscellaneous.

17 studies [10, 15, 17–23, 25–27, 30–35] involving 1,577 knees reported the number of knees with mechanical axis (hip-knee-ankle, HKA axis) malalignment of >3°. The chi-squared test for heterogeneity was 29.15 (*P* = 0.05). Numbers of malalignment were similar in both groups (Figure 4) with risk ratio 0.88 (95% CI, 0.74 to 1.04; *P* = 0.13). According to the radiographic method and PSI system, the subgroup analysis was assigned. For the radiographic subgroup, the result favors the MRI subgroup as CT shows no significant difference (Figure 4). For the PSI system subgroup, there is no significance difference among each subgroup but Visionaire-specific PSI system is the only system that showed significant difference between PSI and CI (Figure 5).

### 3.7. Patient-Reported Outcomes

**Figure 6:**
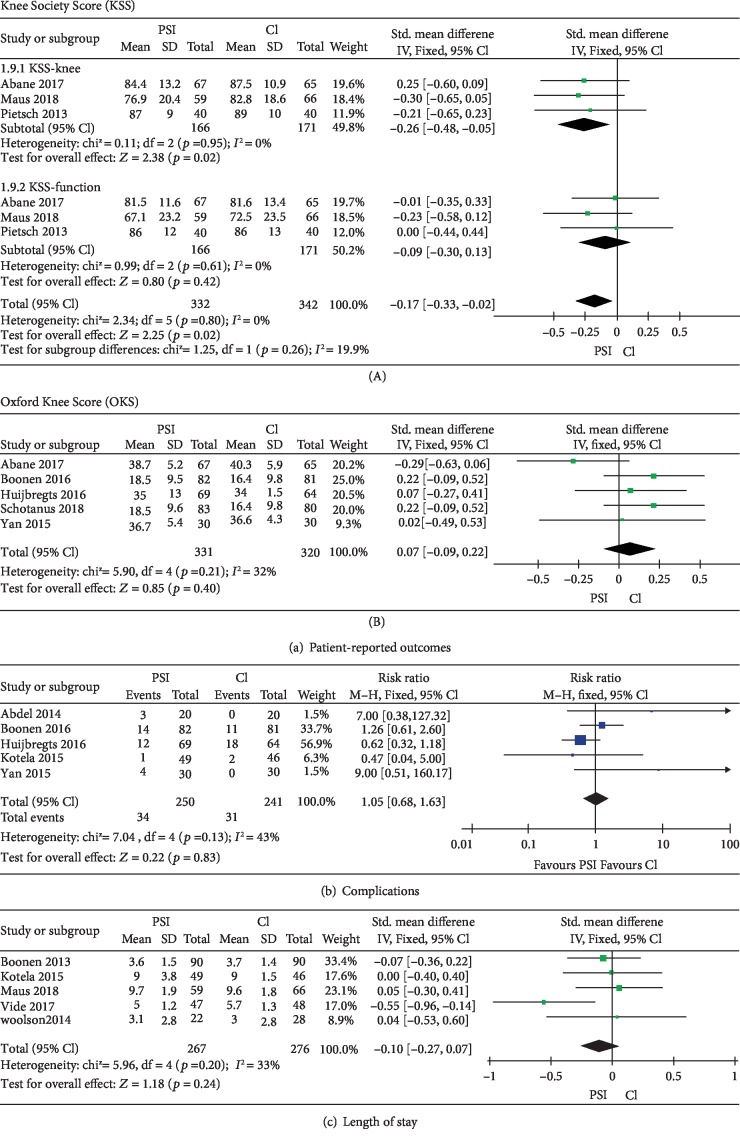
Forest plot of patient-reported outcomes, complications, and length of stay. (a) Forest plot of patient-reported outcomes: (A) Knee Society Score (KSS), subgroup analysis by KSS-knee and KSS-function. (B) Oxford Knee Society (OKS). (b) Forest plot of complications between PSI and CI. (c) Forest plot of length of stay in hospital (days) between PSI and CI. SD: standard difference; PSI: patient-specific instrumentation; CI: conventional instrumentation.

We observed a significant superior outcome of Knee Society Score (KSS) in PSI group compared with CI groups, particularly in KSS-knee group. Patients with the follow-up time of 3 months in 3 studies [12, 15, 24] were included. KSS was analyzed by subgroups of KSS-knee and KSS-function. Meta-analysis was conducted, and the pooled result showed that there is significantly better effect for the PSI group (SMD = −0.17, 95% CI, -0.33 to -0.02, *P* = 0.02). No substantial heterogeneity was found (*I*^2^ = 0%, *P* = 0.02) (Figure 6(a)). For postoperative Oxford Knee Score (OKS), 5 studies [11, 15, 18, 19, 36] were included and no significant difference between PSI and CI groups was found. The total SMD was 0.07 (95% CI, -0.09 to 0.22, *P* = 0.4), and there is no heterogeneity found (*I*^2^ = 32%, *P* = 0.21) (Figure 6(a)).

### 3.8. Postoperative Complication and Length of Stay

Five studies [18, 19, 29, 36, 37] that reported complications were included in meta-analysis (Figure 6(b)) (Table 2) while no significant difference was found (RR = 1.05, 95% CI, 0.68 to 1.63; *P* = 0.83). Superficial surgical site infection/delayed healing was found the most common complication among 29 studies (PSI = 10, CI = 7) (Table 2). Poor range of motion was another common complication observed (PSI = 8, CI = 8).

In the analysis of length of stay, 5 studies [12, 17, 21, 27, 29] were chosen. Fixed effects model meta-analysis confirmed no significant difference for either intervention group (Figure 6(c)).

### 3.9. Publication Bias

**Figure 7:**
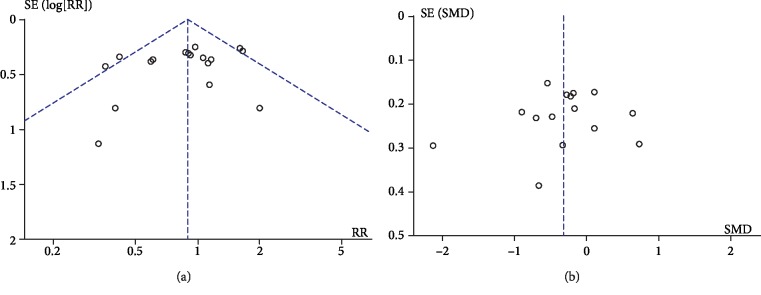
Funnel plots of publication bias. (a) Funnel plot for operative time. (b) Funnel plot for malalignment (hip-knee-ankle axis outliers). SE(log (SMD)): standard error (log (standardized mean difference)); SMD: standardized mean difference; SE(log (RR)): standard error (log (relative risk)); RR: relative risk.

Risk of publication bias in the studies that reported on operative time (15 studies) and malalignment (18 studies) was assessed by graphical assessment of funnel plots (Figure 7). Both plots show minimal evidence of publication bias.

## 4. Discussion

Patient-specific instrumentation (PSI) has been introduced to reduce operation time and increase surgical efficacy due to avoidance of intramedullary canal violation. In our work, we did find a shorter operative time performed by PSI than by CI significantly (SMD = −0.36, *P* = 0.03). Thienpont et al. [8] did a meta-analyses including level 1 and level 2 studies, concluding a slight but significant difference for PSI, consistent with our result. Other studies [5, 38] could not reach a significance perhaps their samples' size was not as large as ours (690 patients for PSI and 714 for CI), thus resulting in the type 2 error.

Compared with the MRI-based group, the CT-based PSI group showed no significance with the CI group, consistent with another study by Noble et al. [28]. In fact, the number of surgical steps of CT-based PSI was not reduced compared with CI, as it did not have a cutting slot or proximal peg holes on the tibial articular surface, which was necessary to use a standard cutting instrument on both the femur and tibia sides [39]. Despite the statistical difference when compared with CI, our subgroup analysis found no significant difference between MRI- and CT-based groups (*P* = 0.66, *I*^2^ = 0).

Besides operative time, significant reduction of blood loss was also observed in PSI group, regardless of calculating as blood volume (*P* = 0.03) or hemoglobin (Hb) count (*P* = 0.0002). This may also be attributed to the minimally invasive operative way of PSI that diminished dissection. None of the three studies [17, 22, 24] considering Hb reduction reached significance as they were limited by separated sample size. We chose a fixed effects model to analyze the Hb while random effects model for the blood volume due to the heterogeneity we found (*I*^2^ = 31% for Hb and *I*^2^ = 71% for blood volume). The higher heterogeneity for blood volume might be due to the different drained time they chose. When we discuss the effect of different instrumentations on the blood loss, hemoglobin is a more ideal variable than blood volume, for it is directly related to the physiological condition and may be of greater clinical relevance.

Overall, with 1500 patients regardless of image acquisition method, we found no difference between two groups regarding outliers of the mechanical axis. While if we compared the subgroups of MRI-based and CT-based with CI groups, we observed a trend of difference toward MRI-based subgroup (*P* = 0.04) than CT-based subgroup (*P* = 0.8). Previous meta-analysis [40] and RCT [32] directly comparing MRI with CT modalities also found slightly lower proportion of outliers in the overall alignment of the limb in MRI groups. However, if we take the safety, cost, and convenience into consideration, the question of superiority becomes far beyond the current research. When analyzing the subgroups with different systems, it appeared that the risk of outlier of the mechanical axis was related to specific systems (Figure 5), consistent with the study of Huijbregts et al. [18].

Evidences currently suggested that malalignment was strongly related to postoperative complications [41], such as patellar tracking [42] and kinematics [43]. PSI was produced to improve the limb alignment of the TKA [11]. Yet, our work did not detect any significant difference between PSI and CI regarding postoperative complication as well as rate of outliers. However, postoperative complication is a multifactorial result and whether those complications were alignment-related or instrumentation-related remains unclear. Boonen et al. [36] and Yan et al. [19], for instance, both got a result that patients in the PSI group actually suffered a higher risk of complication compared with CI groups. A possible reason could be that the surgeons enrolled in this study were high-volume knee arthroplasty surgeons with conventional instrumentation, thus producing a better outcome than the PSI as a newer technique.

Patient-reported outcomes (PROs) are the best subjective measurement of functional outcome after joint arthroplasty [44]. Due to our strict inclusion criteria, only Knee Society Score (KSS) and Oxford Knee Score (OKS) were extracted and analyzed. The difference of OKS is insignificant, consistent with previous reviews conducted by Goyal and Tripathy [7] and Mannan et al. [6]. Unlike their studies, all patients included in our analysis were restricted at a 3-month follow-up. Although data from a long-term outcome could not be extracted, existing RCTs suggest that OKS in 2-year [36] and 5-year [11] follow-up is also statistically not different between PSI and CI.

Although we included all level 1 studies, we still detected risk of bias from some included studies. However, we used strict criteria to assess the quality of studies. For example, studies that were designed with only patients in PSI group receiving the MRI or CT examination were considered as high risk of bias, as the blinding from patients was broken. Besides, studies where surgeons performed TKA but were not independent of the trial were also considered as high risk of performance bias.

Our work has several strengths. Firstly, as a meta-analysis with all level one studies of approximately 2500 patients, it has greater statistical power than all the included studies and the previous published meta-analyses. Secondly, to our knowledge, it is the first review to evaluate intraoperative efficacy, postoperative outcomes (radiology, function, and complications), subgroup analysis (different image acquisition methods and PSI systems used), and length of stay which is strongly related to cost-effectiveness analysis, allowing a more comprehensive appraisal of the PSI technology.

Limitation of our analysis still existed. Firstly, the perioperative procedures between two techniques have seldom been evaluated. Time cost of learning PSI technology and processing the image has not been calculated and analyzed. The minor improvement of surgical efficacy might not offset additional perioperative time [45]. As the learning curve for a new technique (e.g., PSI) has always been a matter of debate, we still need more solid evidences to warrant the indication of patient-specific instrumentations.

## Figures and Tables

**Table 1:** Baseline characteristics of included RCTs.

Study	Sample size		Image acquisition	Age		Follow-up time (month)	Gender (male/female)
	PSI	CI		PSI	CI		
Van 2018	44	50	MRI	67 ± 8.8	64 ± 6.9	24	PSI: 14/30; CI: 18/32
Maus 2018	59	66	MRI	68.1 ± 8.5	71.5 ± 8.1	3	PSI: 26/33; CI: 23/43
Schotanus 2018	83	80	MRI	69 ± 8.0	65 ± 8.8	24	Unspecified
Davide 2018	12	12	Unspecified	74.17 ± 3.71	69.40 ± 7.52	0.1	PSI: 3/9; CI: 2/10
Nienke 2018	21	21	MRI	62.7 ± 4.5	63.4 ± 4.2	12	PSI: 8/13; CI: 12/9
Vide 2017	47	48	MRI	67.8 ± 8.4	69.3 ± 6.5	Unspecified	PSI: 15/32; CI: 15/33
De Vloo 2017	20	24	MRI	72.72 ± 8.89	72.28 ± 7.99	2.7	PSI: 11/14; CI: 10/15
Abane 2017	67	65	CT	69.3 ± 9.6	69.8 ± 9.4	3	PSI: 40/30; CI: 41/29
Huijbregts 2016	69	64	MRI	66.7 ± 9.14	69 ± 9.6	12	PSI: 29/40; CI: 32/32
Boonen 2016	82	81	MRI	69 ± 8.0	65 ± 8.8	57	PSI: 34/56; CI: 40/50
Yan 2015	30	30	MRI	67.5 ± 8	69.5 ± 8.4	3	PSI: 13/17; CI: 6/24
Molicnik 2015	19	19	MRI	67.1 ± 7.1	66.8 ± 6.7	Unspecified	Unspecified
Kotela 2015	49	46	CT	66.1 ± 8.4	68.6 ± 9.9	12	PSI: 16/33; CI: 13/33
Abane 2015	59	67	MRI	67.8	70.4	3	52/88
Woolson 2014	22	26	CT	Unspecified		PSI: 9.5; CI: 10.8	PSI: 22; CI: 26
Victor 2014	61	64	MRI	67	66	Unspecified	PSI: 21/43; CI: 21/43
Silva 2014	23	22	MRI	73	74	Unspecified	Unspecified
Pfitzner 2014	60	30	MRI/CT	65	64	3	PSI: 26/34; CI: 13/17
Chotanaphuti 2014	40	40	CT	69.7 ± 5.5	69.3 ± 5.5	1.4	70/10
Kotela 2014	52	60	CT	66.1 ± 8.4	68.6 ± 9.9	12	PSI: 16/33; CI: 13/33
Abdel 2014	20	20	MRI	71	71	3	PSI: 8/12; CI: 8/12
Roh 2013	42	48	CT	70 ± 7.2	70 ± 5.1	Unspecified	PSI: 3/39; CI: 5/43
Parratte 2013	20	20	MRI	50-85		3	Unspecified
Hamilton 2013	26	26	CT	68.1	67.6	18	PSI: 14/12; CI: 7/19
Boonen 2013	90	90	MRI	69 ± 8.0	65 ± 8.8	24	PSI: 34/56; CI: 40/50
Chareancholvanich 2013	40	40	MRI	69.5	70.3	Unspecified	PSI: 6/34; CI: 4/34
Vundelinckx 2013	31	31	MRI	64.65 ± 8.23	68.19 ± 8.48	7.2	PSI: 15/16; CI: 11/20
Pietsch 2013	40	40	MRI	71.4 ± 6.6	69.2 ± 9.4	2.8	PSI: 13/27; CI: 19/21
Noble 2012	15	14	MRI	65.4	68	Unspecified	PSI: 8/7; CI: 6/8

Abbreviations: PSI: patient-specific instrumentation; CI: conventional instrumentation; CT: computed tomography; MRI: magnetic resonance imaging.

**Table 2:** Complications extracted in PSI group and CI group.

Complications	PSI total	CI total
Superficial surgical site infection/delayed healing	10	7
Poor range of motion	8	8
Manipulation under anesthetic	5	7
Blistering	0	1
Cellulitis	0	1
Geniculate artery pseudoaneurysm	0	1
Haemarthrosis	3	2
Myocardial infarction	0	1
Pneumonia	1	0
Pressure sore	1	0
Pyelonephritis	1	0
Urinary tract infection	0	1
Venous thromboembolism	1	1
Deep infection	0	1
Acute exacerbation of gouty arthritis	1	0
Postoperative flexion contractures	2	0
Preoperative patellar subluxation that continued postoperatively	1	0

Abbreviations: PSI total: patient-specific instrumentation total; CI total: conventional instrumentation total.

## Supplementary Materials

Supplementary 1 S1: PRISMA checklist.

Supplementary 2 S2: Supplemental file: comparison of published reviews with our studies.
