# Supplementary material for: Patient-Specific or Conventional Instrumentations: A Meta-analysis of Randomized Controlled Trials
Source: Biomed Res Int. 2020 Mar 10;2020:2164371. doi: 10.1155/2020/2164371 (PMC7085852; doi:10.1155/2020/2164371)
Supplement: Supplementary 2 — S2: Supplemental file: comparison of published reviews with our studies. [file 2164371.f2.pdf]

Supplementary File. Comparison of published reviews with our studies.

Reviews	Number, publish year(range) and level of evidence of the included studies	Patients included	Variables considered	Subgroup analysis	Conclusion
Alcelik et al. 2017	12<br>1950-2016<br>Level I	PSI: 538<br>CI:549	● **Radiological outcomes: o**utliers for femoral component sagittal, coronal and rotation<br>● al positioning, tibial component sagittal and coronal positioning, overall mechanical axis	NO	Our results suggest that at present PS instrumentation is **not superior** to ST instrumentation in primary total knee arthroplasty.
Mannan et al. 2017	8<br>2000-2015<br>Level 1 :4<br>Level 2: 4	828 knees	● **Functional outcomes:** KSS (function), KSS (knee), ROM, OKS, WOMAC scores.	No	no conclusive evidence for or against PSI when considering short-term functional outcomes. Further high-quality studies are required to investigate both mid- and long-term outcomes as well as survivorship data.
Huijbregts et al. 2016	21<br>2000-2015<br>Level 1 or Level 2	PSI: 805<br>CI: 782	● **radiographic accuracy**<br>● **operation time**<br>● **hospital stay**<br>● **blood loss**<br>● **number of surgical trays required**<br>● **patient-reported outcome measures.**	hip-knee-ankle axis outliers were subgroup analyzed by different patient-specific systems.	Patient-specific instrumentation does not result in clinically meaningful improvement in alignment, fewer outliers, or better early patient-reported outcome measures. Efficiency is improved by reducing **the number of trays used,** but PSI does not reduce operation time.
Goyol et al. 2016	5<br>2000-2015<br>Level 1	379 knees	● **Functional outcomes: KSS, OKS, WOMAC**<br>● VAS (0-10 scale)	No	Current literature is insufficient to address whether there is a benefit of PSI in total knee arthroplasty in terms of improvement in functional outcomes.
Thienpont et al. 2016	44<br>2011-2015<br>Level 1 and Level 2	2866 knees (PSI)2956 knees(CI)	● **radiographic accuracy**<br>● **operation time**<br>● **blood loss**	No	PSI improves the accuracy of femoral component alignment and global mechanical alignment, but at the

			• **patient-reported outcome measures.**		cost of an increased risk of outliers for the tibial component alignment. The impact of the increased probability of tibial component malalignment on implant longevity remains to be determined. Meta-analyses indicated significant differences with regard to operative time and blood loss in favor of PSI. However, these differences were minimal and, by themselves, not a substantial justification for routine use of the technology.
Lin et al.	29<br>2012-2018<br>Level 1	2487 knees<br>PSI: 1243,<br>CI:1244	• **radiographic accuracy**<br>• **operation time**<br>• **hospital stay**<br>• **blood loss**<br>• **rate of complications**<br>• **patient-reported outcome measures.**	1. Mechanical axis outliers were subgroup analyzed by different patient-specific systems and by CT- or MRI-based PSI.<br>2. Operation time was subgroup analyzed by CT- or MRI- based PSI.<br>3. KSS was subgroup analyzed by KSS-knee and KSS-function.<br>4. Blood loss was analyzed by both volume and hemoglobin	1. Generally, PSI did not improve the alignment of mechanical axis compared with CI, but MRI-based PSI and Visionaire-specific PSI could decrease the risk of malalignment significantly.<br>2. PSI could reduce operative time and blood loss and improve the KSS compared with CI, but for CT-based PSI, the difference of operative time become insignificant.<br>3. PSI shown no significant difference with CI regarding risk of complication, length of stay in hospital and functional outcomes of OKS.
